# Geographical distribution and relative risk of Anjozorobe virus (Thailand orthohantavirus) infection in black rats (*Rattus rattus*) in Madagascar

**DOI:** 10.1186/s12985-018-0992-9

**Published:** 2018-05-09

**Authors:** Vololoniaina Raharinosy, Marie-Marie Olive, Fehivola Mandanirina Andriamiarimanana, Soa Fy Andriamandimby, Jean-Pierre Ravalohery, Seta Andriamamonjy, Claudia Filippone, Danielle Aurore Doll Rakoto, Sandra Telfer, Jean-Michel Heraud

**Affiliations:** 1 Virology Unit, Institute Pasteur de Madagascar, Ambatofotsikely, BP 1274 Antananarivo, Madagascar; 2 Ecole Doctorale des Sciences de la Vie et de l’Environnement, Equipe Pathogènes et Diversité Moléculaire, Faculté des Sciences, Université d’Antananarivo, Antananarivo, Madagascar; 3 Plague Unit, Institut Pasteur de Madagascar, Antananarivo, Madagascar; 4 Département de Biochimie Fondamentale et Appliquée, Faculté des Sciences, Université d’Antananarivo, Antananarivo, Madagascar; 5 Institute of Biological and Environmental Sciences, University of Aberdeen, Aberdeen, UK

**Keywords:** Hantavirus, Anjozorobe virus, Thailand orthohantavirus, Rodent, small terrestrial mammals, Risk factors, Madagascar, Africa

## Abstract

**Background:**

Hantavirus infection is a zoonotic disease that is associated with hemorrhagic fever with renal syndrome and cardiopulmonary syndrome in human. Anjozorobe virus, a representative virus of Thailand orthohantavirus (THAIV), was recently discovered from rodents in Anjozorobe-Angavo forest in Madagascar. To assess the circulation of hantavirus at the national level, we carried out a survey of small terrestrial mammals from representative regions of the island and identified environmental factors associated with hantavirus infection. As we were ultimately interested in the potential for human exposure, we focused our research in the peridomestic area.

**Methods:**

Sampling was achieved in twenty districts of Madagascar, with a rural and urban zone in each district. Animals were trapped from a range of habitats and examined for hantavirus RNA by nested RT-PCR. We also investigated the relationship between hantavirus infection probability in rats and possible risk factors by using Generalized Linear Mixed Models.

**Results:**

Overall, 1242 specimens from seven species were collected (*Rattus rattus, Rattus norvegicus, Mus musculus, Suncus murinus, Setifer setosus, Tenrec ecaudatus, Hemicentetes semispinosus*). Overall, 12.4% (111/897) of *Rattus rattus* and 1.6% (2/125) of *Mus musculus* were tested positive for THAIV. Rats captured within houses were less likely to be infected than rats captured in other habitats, whilst rats from sites characterized by high precipitation and relatively low seasonality were more likely to be infected than those from other areas. Older animals were more likely to be infected, with infection probability showing a strong increase with weight.

**Conclusions:**

We report widespread distribution of THAIV in the peridomestic rats of Madagascar, with highest prevalence for those living in humid areas. Although the potential risk of infection to human may also be widespread, our results provide a first indication of specific zone with high transmission. Gathered data will be helpful to implement policies for control and prevention of human risk infection.

## Background

Hantaviruses are zoonotic viruses whose main reservoir hosts are rodents, although these viruses have recently been detected in insectivores and bats [1, 2]. Human infections appear in several areas of the globe. Clinical symptoms and severity of disease vary between regions according to the species of hantavirus involved. Two diseases associated with hantavirus infections are described, the Hantavirus Cardiopulmonary Syndrome (HCPS) and the Hemorrhagic Fever with Renal Syndrome (HFSR). Nephropathia Epidemica (NE) is a mild form of HFSR. While NE disease appears to be mild with 0.1% of mortality rates, HCPS and HFRS, are more severe with a mortality that can reach up to 50 and 15% respectively [3]. HCPS are mainly found in the Americas, and HFRS and NE appears in the Old World [4, 5].

Hantaviruses are generally thought to be relatively host-specific [6], for example, Seoul orthohantavirus (SEOV) is associated with the rat, *Rattus norvegicus*, [7] and Puumala orthohantavirus (PUUV) is preferentially hosted by the bank vole, *Clethrionomys glareolus* [8]. However, studies have shown that spillover infection can occur between sympatric host species [9, 10]. For example, the common vole, *Microtus arvalis*, is the preferential host of Tula orthohantavirus (TULV), but the virus is also detected in other vole species such as *Microtus agrestis* and *Arvicola spec.* [11]. Hantaviruses can be found in saliva, urine and feces of infected animals and transmission between reservoirs is thought to be through bites, scratches or inhalation of contaminated aerosols [12, 13]. Humans usually contract hantavirus infection by direct contact with rodents or by inhalation of contaminated aerosols from excretion or secretion from infected animals [14]. Transmission inter-human is very uncommon, although it has been reported for some lineages of Andes orthohantavirus (ANDV) [15, 16].

Although relatively few hantavirus studies have been carried out in Africa and the surrounding regions, since 2006 eight hantaviruses have been [14] identified in rodents, insectivores and bats in mainland Africa [17–24] whilst recently two hantaviruses were detected in the Indian Ocean region [25, 26]. The two hantaviruses found in the Indian Ocean region both belonged to Thailand orthohantavirus (THAIV) specie which was primarily found in Southeast Asia [27]. Mayotte virus (MAYOV) harbored by *Rattus rattus* in Mayotte island [26] and Anjozorobe virus (ANJOV) found in *Rattus rattus* and the endemic rodent *Eliurus majori* in Madagascar [25].

Studies have also documented exposure of human populations in Africa to hantavirus [28–30], and recently Heinemann et al. have reported the presence of Bowé orthohantavirus associated with human disease in an Ivorian patient [31]. Moreover, as Thailand orthohantavirus is associated with disease [32], it is probable that hantavirus infection in Madagascar is also symptomatic. Nevertheless, the lack of studies in Madagascar prevents us from highlighting the potential threat that this virus presents for public health and from raising awareness amongst clinicians of clinical symptoms associated with hantavirus infection.

In areas of the globe where hantavirus infections in humans are better monitored, there is strong spatial and temporal variation in disease incidence and studies have demonstrated effects of environmental variables, including climate, habitat and land-use [6]. Climatic conditions may influence persistence of hantavirus in the environment and, therefore, transmission rates to humans [33]. However, these environmental effects on incidence are thought to be primarily mediated by the impacts on the abundance of infected reservoirs [34, 35]. For individual hantavirus, spatial variation in risk is likely to largely depend on the bioclimatic and habitat preferences of the principle host, whilst temporal variation in risk may be driven by inter-annual climatic driven changes in food availability and breeding rates [36]. Despite the tendency for close associations between individual mammal species and individual hantaviruses, several studies have also suggested that increased mammal diversity may reduce the prevalence of hantavirus infection in the principal host [37–40], consistent with a “dilution effect”.

For areas where data on infection in humans may be under-recorded, examining the spatial distribution of infected reservoirs provides an important first-step in understanding the potential risk to humans, as well as allowing analyses to explore epidemiological processes in the reservoir populations by identifying environmental risk factors. The previous study in Madagascar that detected Anjozorobe virus was limited to one forest location, Anjozorobe-Angavo forest [25]. Here we sample terrestrial peridomestic small mammals trapped in urban and rural sites of representative regions of Madagascar to (i) establish the distribution of Anjozorobe virus and (ii) evaluate to what extent spatial variation in infection rates is related to environmental and host-related variables. Our results will be helpful to implement policies for future awareness and surveillance programs for human communities, as well as increasing our understanding of the epidemiology of hantavirus in a peridomestic African context.

## Methods

### Specimen collection

Sampling was carried out in twenty districts of Madagascar, with a rural and urban zone in each district except for one district where only the rural zone was sampled. The urban zone was centered on a health center, whilst the rural zone was located within a randomly selected commune within 15 km of the health center. Within the urban zone, three sub-areas for trapping were randomly selected within an 800 m*800 m square, whilst the rural zone centered on a single village. Terrestrial small mammals were captured within a range of habitats. At each zone 15–20 houses were sampled, using one wire-mesh trap (BTS) and one Sherman trap placed inside each house. Where possible a further BTS trap was set in the immediate vicinity of the house. Additional BTS traps were set in trapping lines close to vegetation in (i) areas around houses (either adjacent to paths or areas for disposing of waste) and (ii) around rice fields or other low ground primarily used for agriculture. In urban zones, where possible, additional BTS traps were set in markets and around the abattoir. Traps were baited with onion, dry fish and carrot and checked each morning for 3 days. All trapping was conducted February 2012–April 2012 or October 2012–May 2013. Species, gender and weight of each captured animal were recorded. Animals were euthanized by cervical dislocation and organs were collected and stored in liquid nitrogen before laboratory storage at − 80 °C.

### RNA extraction and nested RT-PCR

Approximately 100 mg of liver and spleen tissues from each individual rodent were ground using Tissuelyser II (Qiagen) with a 5 mm stainless steel beads. Organ supernatants were recovered at 1:10 dilution of culture medium containing 40% of fetal bovine serum and antibiotics. Total RNA was extracted using the TRIzol LS reagents (Invitrogen, Carlsbad, CA) according to the manufacturer’s instructions. cDNA was prepared using PROMEGA Kit One Step PCR and amplified by a previously published nested-PCR protocol that targets conserved region of the L gene [18]. All PCR products were sequenced (Cogenics, Essex, UK).

### Statistical analysis

We investigated the relationship between hantavirus infection probability in rats and possible risk factors by using binomial Generalized Linear Mixed Models (GLMM) with a logit link. Individual infection status was the response. Explanatory covariates were selected for consideration based on their potential to influence transmission of hantavirus within small mammal populations and included individual-level variables and site-level variables. Given that rodents trapped at the same site were non-independent, site was included as a random effect. Continuous covariates were centred and standardized. Non-linear relationships were considered by including the square of the variable. To limit the number of models being considered we performed statistical analysis in two stages: first examining individual-level variables and then site level variables. Model selection was based on likelihood ratio tests (LRT) to compare nested models, and Akaike Information Criteria (AIC) to compare non-nested models. Models with an AIC within 2 of the model with the lowest AIC (ΔAIC< 2) are equally likely to be the best model [41].

The individual-level stage of the analysis considered weight (as a proxy for age), sex and habitat. Two different habitat related variables were considered. The first was a six-level factor including inside house, outside house, abattoir, market, exterior trap lines and low-ground trap lines. The second was a two-level factor that compared inside house rats to rats from all other habitats mentioned above. Explanatory variables were first considered in univariate models. However, initial analyses indicated that weight had a very strong effect and models without weight sometimes had problems converging, weight was therefore included in all models.

The site-level stage of the analysis included all variables significant from the first stage of the analysis and added site-level variables individually. A range of variables related to climate were considered. Bioclimate was classified into four zones: dry, subarid, subhumid and humid. The following climatic variables were also obtained from WorldClim (http://worldclim.org/version2): Annual Mean Temperature BIO1, Mean Diurnal Range BIO2, Temperature Seasonality BIO4, Annual Precipitation BIO12, Precipitation Seasonality BIO15 and Precipitation of Driest Quarter BIO17. WorldClim variables were extracted from a 10 km radius from a point mid-way between the urban and rural sites. As these climatic variables were correlated, it would not be possible to consider them together in subsequent multivariate GLMMs. We therefore also used principle components analysis (PCA) to summarize climatic variation between sites and considered the first two principal components as covariates in the GLMM. In addition to climatic variables, we also examined site type (rural vs urban); season which was defined as dry season October to December and rainy season January to April; inside house *R. rattus* abundance (number rats trapped in houses/number of trap nights in houses), outside house *R. rattus* abundance (number rats trapped outside/number of trap nights outside); host species diversity based on the Shannon diversity index and host species diversity based on Evenness [42].

In each stage, following individual assessment of variables, non-correlated variables with a *p*-value for the LRT of < 0.20 were considered for inclusion in a multivariate model. Statistical analyses were conducted in R software version 3.3.1 using the following packages: vegan, ade4 and lme4 [43–46].

## Results

Seven species of small mammal were trapped. *R. rattus* were trapped in nearly all (97%) of the sites (38/39), whilst *R. norvegicus* were primarily trapped in the large cities (31% of sites, 12/39). We tested specimens from 1242 mammals belonging to seven species: 897 black rats *R. rattus*, 125 house mouse *Mus musculus*, 124 Norway rats *R. norvegicus* and 96 insectivores (*Suncus murinus, Setifer setosus, Tenrec ecaudatus, Hemicentetes semispinosus*) (Table 1). The number of animals tested per site ranged from 11 to 62.

**Table 1:** Prevalence of hantavirus nucleic acids detected in small terrestrial mammals captured in Madagascar

District	n/N of captured species (%)							n/N (%)
	*Rr*	*Rn*	*Mm*	*Sm*	*Ss*	*Hs*	*Te*	
Antsirabe	3/61 (4.9)	0/9 (0.0)	0/7 (0.0)	0/2 (0.0)	–	–	–	3/79 (2.7)
Miandrivazo	9/70 (12.9)	–	0/7 (0.0)	0/3 (0.0)	–	–	–	9/80 (8.0)
Ihosy	1/58 (1.7)	–	0/12 (0.0)	–	0/2 (0.0)	–	–	1/72 (0.9)
Nosy-Be	3/24 (12.5)	0/17 (0.0)	0/1 (0.0)	0/13 (0.0)	–	–	–	3/55 (2.7)
Antsohihy	4/34 (11.8)	0/6 (0.0)	0/3 (0.0)	0/11 (0.0)	0/2 (0.0)	–	–	4/56 (3.5)
Toamasina	6/23 (26.1)	0/12 (0.0)	0/2 (0.0)	0/10 (0.0)	0/3 (0.0)	–	0/1 (0.0)	6/51 (5.3)
Sambava	5/86 (5.8)	–	–	0/3 (0.0)	–	–	–	5/89 (4.4)
Mandritsara	15/61 (24.6)	–	0/5 (0.0)	–	–	–	–	15/66 (13.3)
Morombe	3/57 (5.3)	–	2/3 (66.7)	–	–	–	–	5/60 (4.4)
Morondava	1/17 (5.9)	0/29 (0.0)	0/5 (0.0)	0/2 (0.0)	–	–	–	1/53 (0.9)
Belo sy Tsiribihina	0/31 (0.0)	0/11 (0.0)	0/10 (0.0)	0/4 (0.0)	0/4 (0.0)	–	–	0/60 (0.0)
Fianarantsoa	16/60 (26.7)	–	0/9 (0.0)	0/1 (0.0)	–	0/1 (0.0)	–	16/71 (14.2)
Farafangana	13/55 (23.6)	–	0/2 (0.0)	0/8 (0.0)	–	–	0/2 (0.0)	13/67 (11.5)
Mananjary	5/53 (9.4)	–	0/3 (0.0)	0/6 (0.0)	0/4 (0.0)	–	–	5/66 (4,4)
Antananarivo- Atsimondrano	7/30 (23.3)	–	0/10 (0.0)	–	–	–	–	7/40 (6,2)
Maevatanana	11/44 (25.0)	0/1 (0.0)	0/2 (0.0)	0/8 (0.0)	–	–	–	11/55 (9,7)
Moramanga	4/14 (28.6)	0/24 (0.0)	0/11 (0.0)	0/1 (0.0)	–	–	–	4/50 (3,5)
Ambovombe-Androy	0/57 (0.0)	–	0/16 (0.0)	–	–	–	–	0/73 (0.0)
Taolagnaro	1/27 (3.7)	0/9 (0.0)	0/4 (0.0)	0/2 (0.0)	–	–	–	1/42(0.88)
Anjozorobe	4/35 (11.4)	0/6 (0.0)	0/13 (0.0)	0/2 (0.0)	–	0/1 (0.0)	–	4/57(3.54)
TOTAL	111/897 (12.4)	0/124 (0.0)	2/125 (1.6)	0/76 (0.0)	0/15 (0.0)	0/2 (0.0)	0/3 (0.0)	113/1242 (9.1)

*Rr Rattus rattus, Rn Rattus norvegicus, Mm Mus musculus, Sm Suncus murinus, Ss Setifer setosus, Hs Hemicentetes semispinosus, Te Tenrec ecaudatus*, *N* total number of samples tested, *n* number of hantavirus positive samples, *%* hantavirus prevalence

**Fig. 1:**
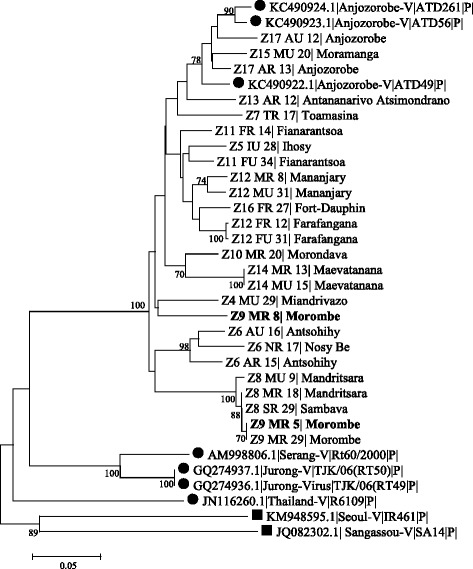
Phylogenetic tree of hantavirus based on the partial sequences of the L segment coding region. Phylogenetic tree generated by the Neighbour-joining methods and Kimura-2 parameter, based on the alignment of the coding region of the L partial segment 301 nucleotides long of rodents-borne hantaviruses detected in Madagascar. ●Reference Sequences ■ Outgroup sequences obtained from Genbank (http://www.ncbi.nlm.nih.gov/nuccore/. Our strains are indicated by Z and the initial of the district followed by the name of the isolation site. In bold are the sequences of hantavirus detected in *Mus musculus*. Only bootstrap percentages ≥70% (from 1000 resampling) is indicated. Scale bar indicates nucleotide substitution per site

Overall 113 (9%) small mammals were PCR positive for hantaviruses, of which 111 were *R. rattus* and two *M. musculus*. It is to be noted that all PCR-positive samples were confirmed by sequencing. Hantavirus RNA was not detected in samples from *R. norvegicus* or insectivores. Genetic analysis of the partial the coding region of the L segment (301 nt) of rodent-borne hantaviruses detected in Madagascar show that these viruses cluster with previous hantavirus detected in Madagascar; Anjozorobe virus (Fig. 1).

**Fig. 2:**
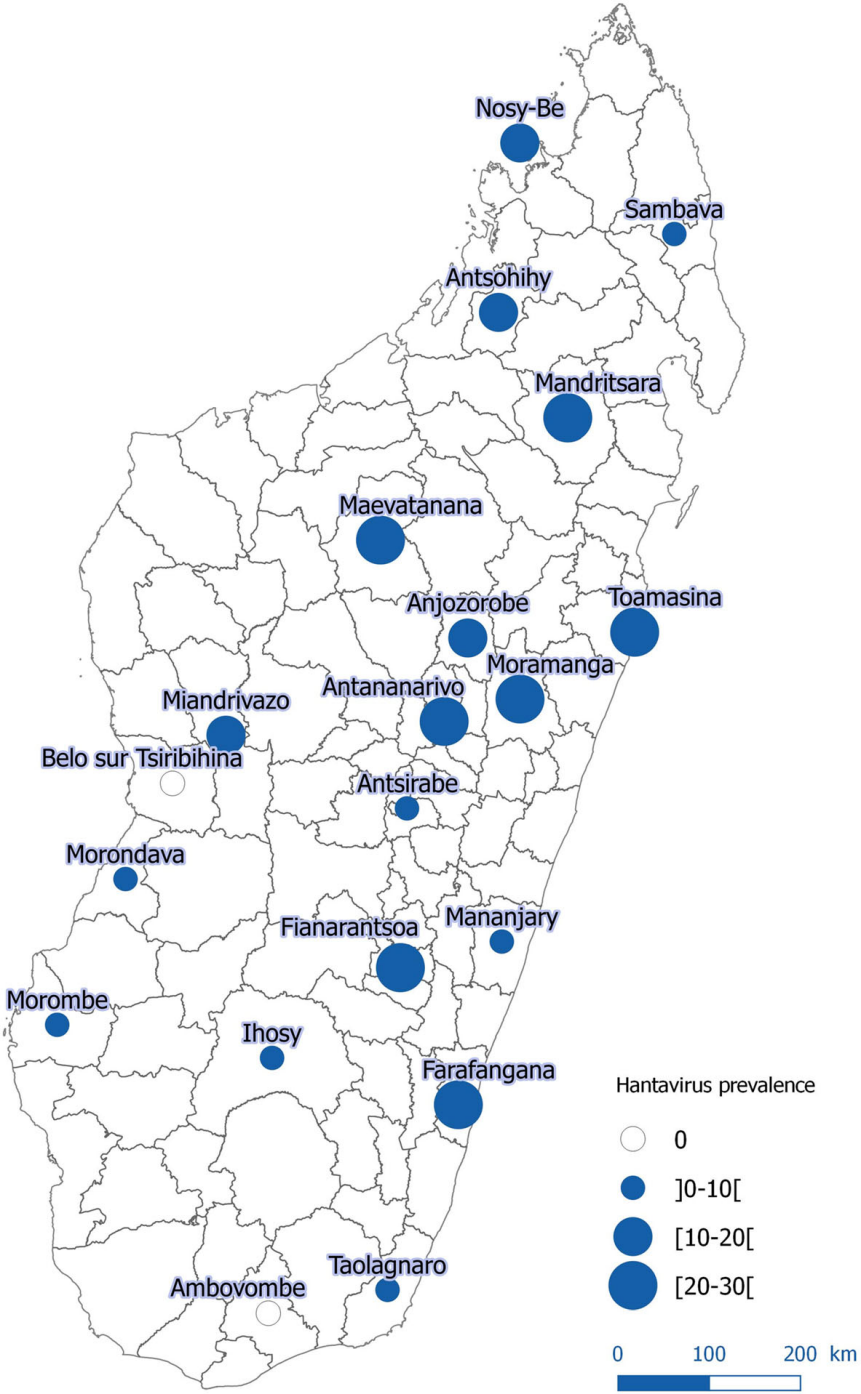
Geographical distribution of hantavirus detected in small terrestrial mammals trapped in Madagascar

Positive individuals showed a widespread geographical distribution, with infected individuals in 90% of districts (18/20) (Fig. 2) and 69% of sites (27/39). The prevalence of infected *R. rattus* at district level ranged from 0 to 29% (Table 1). The highest prevalences were obtained in the districts of Moramanga (29%) Fianarantsoa (27%), Toamasina (26%), Maevatanana (25%), Mandritsara (25%), Farafangana (24%) and Antananarivo-Atsimondrano (23%).

At the site level, prevalence in *R. rattus* ranged from 0 to 80%, with highest prevalences in Moramanga Urban (80%, *n* = 5), Mandritsara Rural (58%, *n* = 19), Fianarantsoa Urban (43%, *n* = 30) and Anjozorobe Urban (43%, *n* = 7). For the 19 sites where both urban and rural sites were tested there was no correlation between the prevalence in the urban and the rural site (Spearman rank, *p = 0.9*).

### Risk factors of hantavirus infection in *R. rattus*

For these analyses, only *R. rattus* were considered due to the small number of hantavirus positive from the other species.

In the individual-level stage, univariate logistic analyses indicated that weight was an important factor with a highly significant positive linear effect on hantavirus infection probability (Table 2). There was no difference between males and females. However, significant differences in infection probabilities were observed between habitats. Based on Akaike information criterion (AIC), the model with habitat as a two-level factor appears to sufficiently describe this variation, with houses having a lower probability of infection than other habitats (Table 2). Prevalence of infected rodents living within houses or outside were 7.5% (34/456) and 17.5% (77/441) respectively.

**Table 2:** Estimated parameters from individual-levels variables of *Rattus rattus* based on Generalized Linear Mixed Models

Variable		Models	OR	CI 95%	AIC^a^	LRT	*p*_value
Weight		Linear	2.43	1.84–3.23	575	41.72	< 0.001
		Non-Linear	0.96	0.78–1.18	576.8	0.15	0.7
Sex ^a^			0.75	0.47–1.22	575.6	1.36	0.25
Habitat ^a^	House		1		573.28	11.68	0.04
	Outside house		2.87	0.87–9.49			
	Abattoir		0.97	0.22–4.28			
	Market		2.32	0.94–5.72			
	Exterior line-trap		1.89	0.93–3.84			
	Low-ground		2.68	1.36–5.28			
Habitat ^a^	House		1		567.9	9.04	0.003
	Exterior		2.19	1.3–3.69			

Variable Habitat: where animal was caught in house and exterior including outside house, Abattoir, market, Exterior line-trap, area around rice fields or other low ground with vegetation. Variables in bold had significant effect on hantavirus infection with *p*_value< 0.05

*OR* Odds Ratio, *CI* Confidence interval, *AIC* Akaike information criterion

^a^All models included a linear effect of weight due to convergence issues (see text)

**Fig. 3:**
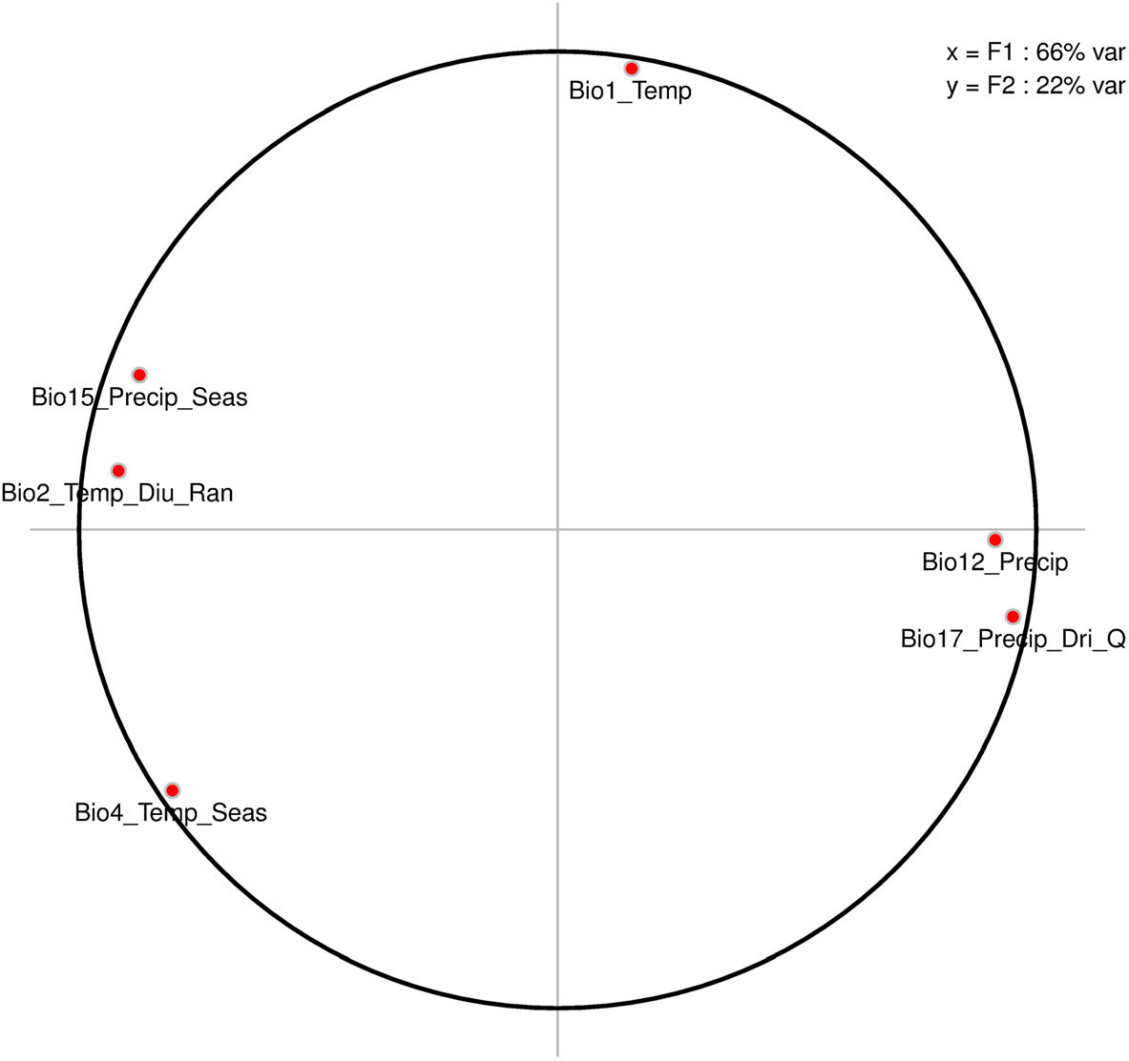
Correlation between the first two principal components and the six climatic variables. Bio 1: Annual Mean Temperature, Bio 2: Mean Diurnal Range in temperature, Bio 4: Temperature Seasonality, Bio12: Annual Precipitation, Bio 15: Precipitation Seasonality, Bio 17: Precipitation of Driest Quarter. Axis x or F1 and Axis y or F2 represented 66 and 22% of the climatic data information respectively. F1 was positively correlated with Bio 12 and Bio 17. However, F2 was negatively correlated with Bio 2 and Bio 15

Principal Components Analysis (PCA) results showed that the two principal components F1 and F2 represented 88% of the total climatic data information (Fig. 3). F1 was positively correlated with annual precipitation and precipitation of driest quarter and negatively correlated with mean diurnal range in temperature and precipitation seasonality. Thus, humid sites were characterized by high values of F1, whilst subarid and some subhumid sites that showed more seasonal variation had low values of F1. F2 exhibited positive correlation with mean annual temperature (Fig. 3), with dry sites having the highest values for F2.

The GLMMs in the site-level stage showed strong evidence of climatic effects. In models that included the best model from the individual-level stage and single site-level covariates, the mean diurnal ranges in temperature, annual precipitation and F1 from the PCA analysis were all associated with variation in infection probability based on LRT (Table 3). There was evidence of a non-linear relationship with F1 and some suggestion of a non-linear effect for mean diurnal range in temperature and annual precipitation (Table 3). Infection probabilities increased with increasing precipitation (OR = 2) and increasing values of F1 (at least to some kind of plateau) and decreased with an increasing diurnal range in temperature (OR = 0.56). There was no evidence of any difference between rural and urban sites. There was also no effect of season, rodent abundance measures or host diversity measures. Consequently, as the climate measures were highly correlated no further multi-variate models were considered.

**Table 3:** Effect of site-level independent variables of *Rattus rattus* hantavirus infection: comparison of linear and non-linear Generalized Linear Mixed Models

Variable		Model	OR	CI 95%	LRT	DF	*p*-value	AIC
No additional variables (weight + habitat)								**567.9**
Bioclimat	Subarid		1		5.5	3	0.14	568.4
	Dry		7.15	1–51.26				
	Humid		8.34	1.22–56.83				
	Subhumid		6.24	1–38.79				
Season			1.24	0.39–3.88	0.13	1	0.72	569.8
Rural/Urban			1.03	0.35–2.97	0.002	1	0.96	570.2
*Rattus rattus* (*Rr*) abundance	*Rr* caught within houses	Linear	0.83	0.51–1.36	0.53	1	0.47	569.4
		Non-linear	1.22	0.78–1.93	0.72	1	0.4	570.7
	*Rr* caught outside trap lines	Linear	1.18	0.7–1.99	0.39	1	0.53	569.5
		Non-linear	1.08	0.62–1.9	0.08	1	0.73	571.5
Shannon diversity index		Linear	1	0.63–1.59	0.00	1	1	569.9
		Non-linear	1.08	0.62–1.9	0.21	1	0.65	571.7
Evenness diversity index		Linear	0.84	0.49–1.41	0.45	1	0.5	569.5
		Non-linear	0.92	0.58–1.47	0.12	1	0.73	571.45
Mean annual temperature		Linear	1.11	0.66–1.87	0.15	1	0.7	569.8
		Non-linear	1.09	0.61–1.95	0.09	1	0.76	571.7
Mean diurnal range in temperature		Linear	0.56	0.33–0.96	4.53	1	**0.03**	565.4
		Non-linear	0.58	0.31–1.09	3.19	1	*0.07*	564.2
Temperature seasonality		Linear	0.6	0.36–1.01	3.67	1	*0.06*	566.3
		Non-linear	0.6	0.28–1.26	1.79	1	*0.18*	566.5
Annual precipitation		Linear	2	1.28–3.12	8.67	1	**0.003**	**561.3**
		Non-linear	0.75	0.54–1.04	3.22	1	*0.07*	**560.0**
Precipitation seasonality		Linear	0.8	0.47–1.36	0.67	1	0.41	569.3
		Non-linear	1.02	0.53–1.97	0.004	1	0.95	571.3
Precipitation of driest quarter		Linear	1.45	0.89–2.37	2.16	1	*0.14*	567.8
		Non-linear	1.05	0.49–2.23	0.02	1	0.9	569.7
PCA Axis 1 (F1)		Linear	1.31	1.02–1.68	4.36	1	**0.04**	565.6
		Non-linear	0.82	0.69–0.98	5.21	1	**0.02**	562.4
PCA Axis 2 (F2)		Linear	1.08	0.71–1.66	0.13	1	0.72	569.8
		Non-linear	1.16	0.8–1.68	0.61	1	0.43	571.2

Weight and Habitat (significant variables from Table 2) were included in each model. For non-linear models, likelihood ratio tests (LRT) compare the non-linear model with the linear model. For linear models, LRT compare the linear model with the best model from Table 2 (includes weight and two-level habitat variable). *P*-values < 0.05 are shown in bold; *p* values > 0.05 and < 0.20 are shown in italics. AIC values within 2 of the model with the lowest AIC are shown in bold

*OR* Odds Ratio, *CI* Confidence interval, *AIC* Akaike information criterion, *LRT* likelihood ratio tests, *DF* Degree of Freedom, *PCA* Principal Components Analysis

Comparison of AIC values from the univariate models indicated that models including precipitation performed best (Table 3). Although, the model with the lowest AIC included a non-linear effect of precipitation, a simple linear effect was similar in its ability to describe the data. The model with a non-linear effect of F1 was marginally worse at explaining the data (ΔAIC = 2.32). Thus, high precipitation appears to be the principle climatic driver of high hantavirus infection probability.

## Discussion

Our national-scale study demonstrates that THAIV is widespread in the peridomestic small mammal communities of Madagascar, primarily infecting *R. rattus*, the most common species in our study, in both rural and urban sites. Rats living outside houses are more likely to be infected than those inhabiting houses and hantavirus prevalence is higher in humid areas of the island. Weight also influenced infection probability, as several studies have shown before for this chronic infection [47, 48]. Our results are important for understanding the potential risk to humans from hantavirus infection in Madagascar.

We confirm *R. rattus* as the main reservoir of hantavirus in Madagascar. Sequencing results from a subset of samples form a distinct cluster with Anjozorobe virus. The detection of two infected *M. musculus* may suggest spillover infections. Spillover infections in sympatric hosts of closely related species or genera have been reported before for Dobrava orthohantavirus (DOBV) [49] and Tula orthohantavirus (TULV) [11]. Our results are consistent with the idea of that whilst hantaviruses have preferred host species, some spillover infections can occur [11]. However, the importance of such secondary spillover hosts for the persistence and dynamics of infection is unclear. For TULV, the spatial distribution of infected individuals from secondary host species and their apparent clearance of the virus suggest they play a minor role, if any [11].

We did not detect any hantavirus in two other introduced mammals, *R. norvegicus* and *S. murinus*. *R. norvegicus* is the main reservoir of Seoul orthohantavirus, a virus that is found predominantly in the Old World [50–52], but thought to have a more widespread global distribution [2] and recently confirmed using molecular techniques as causing human infections in Europe [53, 54]. *S. murinus* has been found infected with Thottapalayam orthohantavirus in India, Thailand and Indonesia. As our sample size for *R. norvegicus* and *S. murinus* were relatively small (*n* = 124 and *n* = 76 respectively), we cannot exclude the possibility that they are infected with hantavirus in Madagascar.

The global prevalence of infection amongst *R. rattus* was 12.4% (111/897), which is similar to the prevalence of Mayotte virus observed in *R. rattus* populations on Mayotte Island (18.1%: 29/160) [26]. However, we detected variation in prevalence at two spatial scales. Large-scale spatial variation was not driven by any rural-urban split but was related to climate, whilst there was also within-site variation related to habitat type. Spatial prevalence patterns at both scales may be associated with variation in transmission rates due to changing contact rates between potential hosts and infective particles. Serological infection may have found more rats that have been exposed to hantaviruses, but using a more conservative test that detects current presence of DNA is arguably more relevant for the potential infection risk for humans as these individuals are actively infected, moreover uncertainty about specificity of serological testing and the potential for cross-reaction with antibodies against other viruses could have resulted in noise within the dataset (false positives). Furthermore, RT-PCR could allow the detection of new variant of ANJOV or a new orthohantavirus which was of importance for us since to date, hantavirus described in Madagascar, were detected from *R. rattus* collected in a small region of Madagascar [25].

At the national scale, sites in humid environments presented the highest rates of hantavirus infection. Climatic drivers, including increased precipitation, have been linked to inter-annual variation of hantavirus cases in humans in China and the Four Corners Region of the USA [33, 34, 55]. Various mechanisms for these relationships have been proposed, including increased vegetation growth leading to more abundant rodent populations and increased contact and viral transmission between rodents and between rodents and humans [34, 55]. A positive relationship between host abundance and infection prevalence has been found for Puumala orthohantavirus in a spatiotemporal study of bank vole populations [39]. However, some other studies have found only weak relationships between principal host abundance and prevalence [56], and in our study we did not observe any significant association between rat abundance and hantavirus prevalence. The absence of strong effects of host abundance may reflect difficulties in measuring abundance in a relevant way, for example current prevalence may depend on previous host abundance rather than current host abundance, or because the relationship between abundance and contact rates is not linear [57]. Alternatively, other factors may drive the association between high precipitation and high prevalence through impacts on contact rates.

Rather than absolute rat abundance, the positive association may instead be linked to variation in the seasonality of reproduction. As indicated by the PCA analysis, sites with high precipitation are also characterized by less seasonality. Rats inhabiting such environments, where there are sufficient food resources throughout the year to allow reproduction, may exhibit behaviors that are linked to hantavirus transmission throughout the year (e.g. aggressive behaviors or behaviors associated with maintenance of territories). As humidity can maintain the infectivity and the stability of virus in the ex-vivo environment [58], an alternative explanation could be that increased contact between rodents and infective particles increases hantavirus infection rates in humid areas.

Within sites, rats trapped outside were more likely to be infected than rats trapped inside houses. This variation could also be due to differences in rat contact structures and virus persistence. Contact structures between rats living in houses are likely to be very different from rats inhabiting outdoors. If rats typically inhabit a single house, each house may represent a single small and relatively isolated population with a much-reduced contact structure. A non-exclusive alternative hypothesis could be that persistence of hantavirus in the environment is reduced within houses.

Several studies of hantavirus in reservoir populations have found an apparent dilution effect, with lower prevalence in sites with increased mammal diversity [37–39]. Such an effect can occur through a variety of mechanisms such as decreases in host density due to competition or reductions in encounter rates between infected and susceptible hosts. In studies of Sin Nombre virus there has been some evidence that increased mammal diversity leads to reduced intraspecific contact rates in the principal host species [37]. In our study we found no evidence of an effect of diversity, measured at the site-level, on hantavirus prevalence and it seems unlikely that community diversity explains the difference in infection probability between inside and outside rats, as all the species trapped were caught in both types of locations (apart from *M. musculus*, which could only be captured by Sherman traps which were restricted to houses, and the rarely trapped *H. semispinosus*). The lack of a dilution effect in our study may suggest that whilst an effect of community composition appears to be common for hantaviruses in reservoir populations inhabiting natural and semi-natural habitats located in several parts of the world [59], it is not a feature of hantaviruses in a peridomestic context. Alternatively, as for abundance, our measures of community diversity may be inappropriate to capture the underlying mechanism. For example, the effects of community composition on Puumala orthohantavirus were driven by abundance of a specific species, rather than overall community diversity [39, 60]. Thus, more detailed analyses, as well as data from more diverse sites, would further improve our understanding of the potential mechanisms behind the spatial variation in prevalence.

## Conclusions

To conclude, we report the widespread distribution of THAIV in the peridomestic rat*, R. rattus*, captured in both urban and rural sites of Madagascar, with highest prevalence in humid areas of the island. Thus, although the potential risk of transmission to humans may also be widespread, our results provide a first indication of areas where the risk may be higher. The reduced infection probabilities in rats living in houses may decrease the risk of transmission to humans, but data on human exposure is necessary to properly evaluate the risk.
